# The sequelae of a missed Essex-Lopresti lesion

**DOI:** 10.1007/s11751-013-0153-z

**Published:** 2013-02-08

**Authors:** K. Thomason, K. J. Burkhart, K. Wegmann, L. P. Müller

**Affiliations:** 1 Robert Jones and Agnes Hunt Orthopaedic Hospital NHS Foundation Trust, Oswestry, Shropshire SY10 7AG UK; 2 Department for Orthopaedic and Trauma Surgery, University Hospitals of Cologne, Cologne, Germany; 3 Klinik und Poliklinik für Orthopädie und Unfallchirurgie, Universitätsklinikum Köln, University Hospitals of Cologne, Kerpener Str. 62, 50937 Cologne, Germany

**Keywords:** Essex-Lopresti, Radial head fracture, Radial head resection, Complication, One bone forearm

## Abstract

Radial head fractures are the most common type of elbow fracture in adults. Unrecognised disruption of the intraosseous membrane at the time of injury can lead to severe wrist pain from proximal radial migration especially if the radial head is excised. In this case, despite anatomical reduction and internal fixation of the radial head fracture, longitudinal forearm instability developed after delayed radial head resection was performed 7 months post-injury. A Suave-Kapandji procedure was performed due to ongoing wrist pain. Because of the previous radial head resection, this led to a floating forearm that could only be solved by creating a one-bone forearm, sacrificing all forearm rotation to achieve a stable lever arm between the elbow and wrist joint.

## Introduction

We describe a case of refractory forearm instability caused after an unrecognized injury to the interosseous membrane (IOM) following a Mason II radial head fracture. The fracture was treated primarily with open reduction and internal fixation. Having failed to diagnose the Essex-Lopresti lesion at presentation, delayed proximal radial migration occurred 7 months after the index injury when the radial head was resected due to ongoing pain at the elbow. Worsening forearm instability led to multiple operations culminating in the creation of a radio-ulnar synostosis, sacrificing all forearm rotation to achieve a stable lever arm between the elbow and wrist joint.

## Case report

In November 2005, a 31-year-old right hand–dominant nurse sustained a left Mason II radial head fracture after a fall which was treated with open reduction and internal fixation using a radial head plate.

**Fig. 1:**
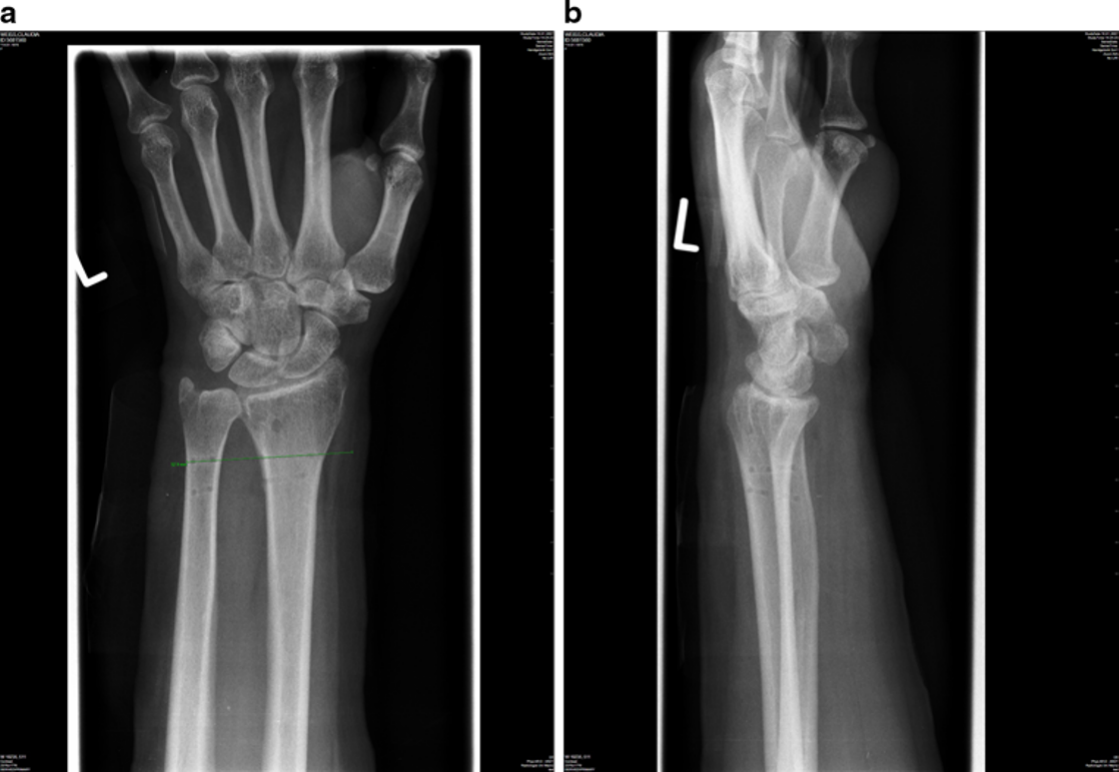
AP and lateral wrist radiographs showing proximal radial migration

Despite regular physiotherapy, the patient complained of pain and stiffness at the elbow. At 6-month post-injury, a CT of the elbow showed degeneration of the radial head so in June 2006, the radial head was resected and a capsular release of the elbow was performed. Soon after, the patient reported worsening pain and instability at the wrist and elbow. Post-operative radiographs revealed proximal radial migration (Fig. 1). A wrist MRI revealed a rupture of the triangular fibrocartiliage complex (TFCC) and a dorsally subluxed distal radioulnar joint (DRUJ).

**Fig. 2:**
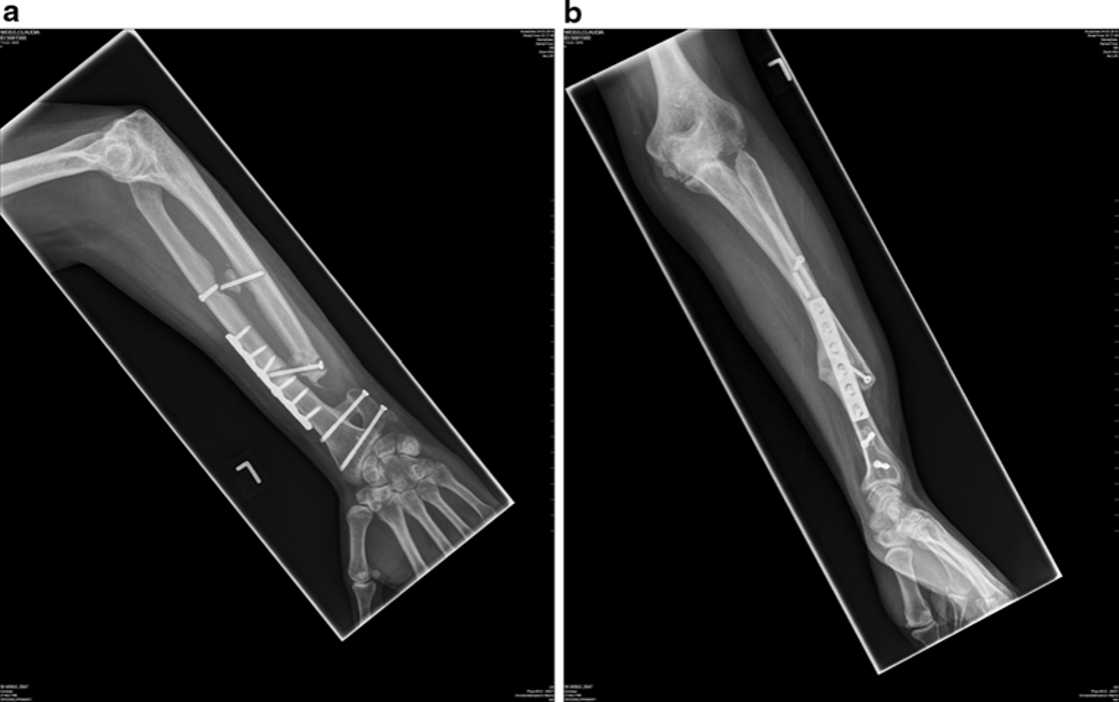
AP and lateral forearm radiographs showing failed synostosis

A soft tissue stabilization of the DRUJ was performed using Palmaris longus tendon as described by Adams [1]. Unfortunately, forearm function and pain on rotation failed to improve and after a further year of ongoing symptoms, a Suave-Kapandji procedure was performed. No symptomatic benefit was achieved and the evidence suggested the presence of complete longitudinal radio-ulnar dissociation or floating forearm. After several discussions with the patient, the decision was made to perform a surgical radioulnar synostosis. Autologous graft from the iliac crest was held in position with two 3.5-mm cortical screws and temporary stabilizing k-wires were removed after 3 weeks. Unfortunately, after 1 month, the radial shaft fractured at the level of one of the transfixing screws and required plate fixation. Over several months, further evidence of a failed synostosis became apparent with resorption of the bone graft, screw breakage and worsening pain, and instability (Fig. 2).

**Fig. 3:**
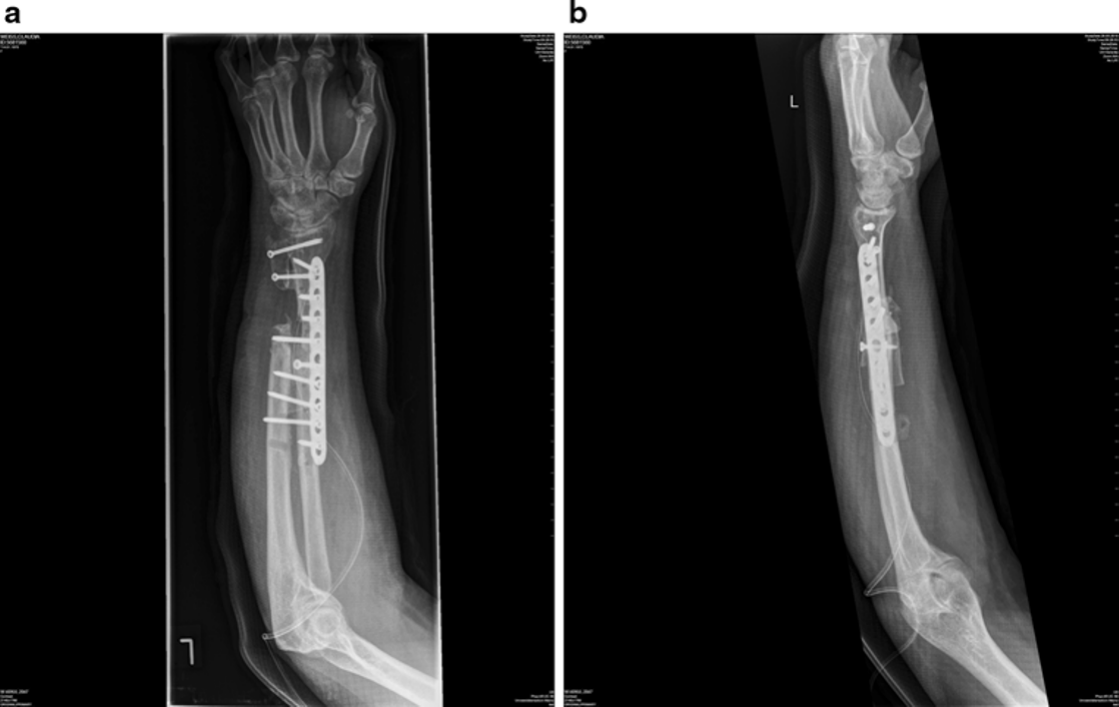
AP and lateral forearm radiographs after revision synostosis

**Fig. 4:**
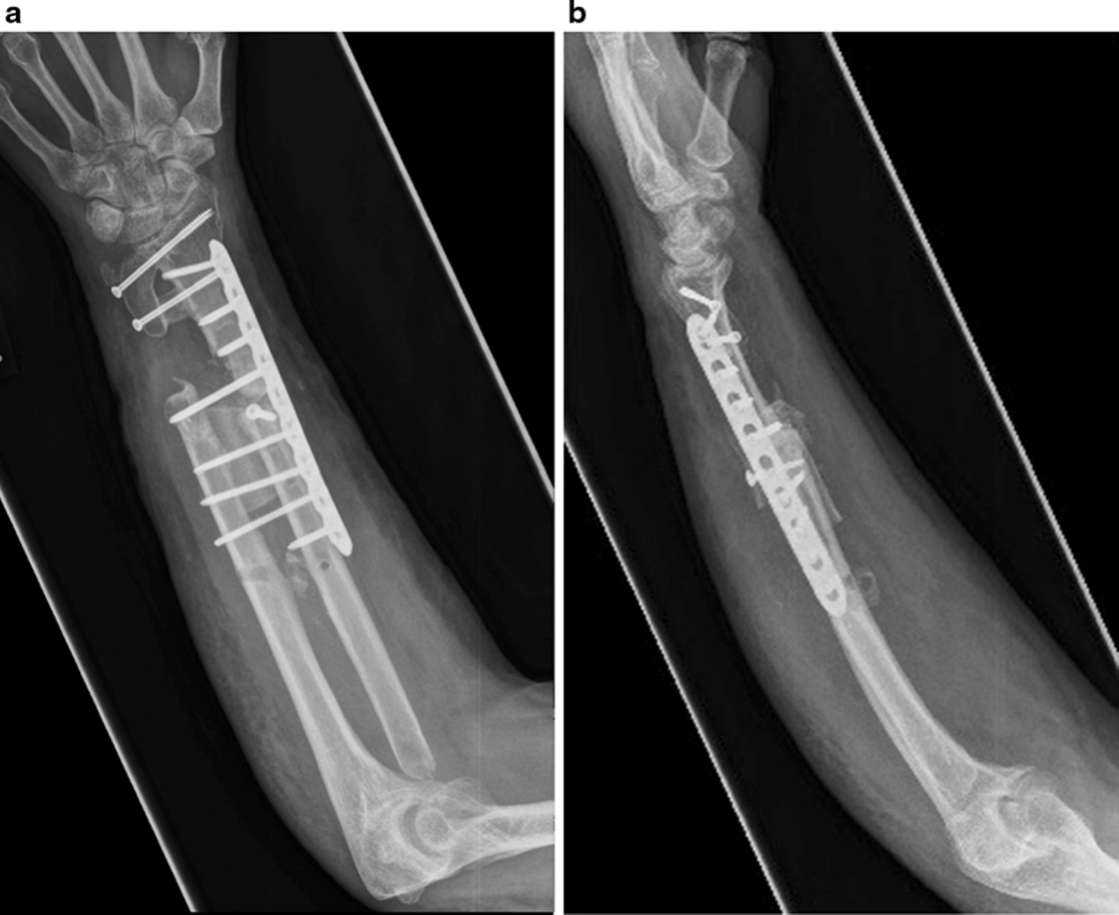
AP and lateral forearm radiographs of the healed one-bone forearm

A revision of the non-union was performed 9 months later with replating of the radius, bulk tricortical iliac crest bone grafting and cortical screw augmentation (Figs. 3, 4). At the patient’s request, the forearm was fixed in supination.

## Discussion

Traumatic rupture of the forearm interosseous membrane with or without associated fracture has been considered a rare injury, although there is evidence to suggest it happens much more frequently than expected [2].

Longitudinal stability of the forearm requires an intact radial head, an intact triangular fibrocartilage complex and an intact interosseous membrane. Essex-Lopresti first described the unhappy triad of radial head fracture, acute DRUJ dislocation and tear of the IOM in 1951 [3] and since then several variants have been described, with or without radial head fracture [4, 5].

The traditional method of treatment in the acute situation has been anatomical restoration of the radial head and reduction of the DRUJ followed by prolonged forearm immobility. Recently, there have been several reports casting doubt on the ability of the torn interosseous membrane to heal by this method [6, 7] especially in the presence of anterior compartment muscle herniation [8].

Chronic interosseous membrane insufficiency is acknowledged to be a much more challenging problem to manage and a variety of methods have been described to repair or reconstruct the central band of the membrane using tendon grafts such as pronator teres [9], flexor carpi radialis [10], semitendinosus [11], palmaris longis [12], Achilles’ tendon [13, 14] or using a bone-patellar tendon-bone graft technique [15]. Ideally, the aim is to re-establish the integrity of the forearm stabilizers with interosseous membrane and TFCC reconstruction whilst levelling the DRUJ to eliminate the ulnar-sided wrist pain caused by the positive ulnar variance.

Although the indications are very rare, the creation of a single bone forearm (SBF) using bicortical bone blocks from the iliac crest is a well accepted means of addressing refractory forearm instability in the salvage situation. All forearm rotation is sacrificed to achieve a stable osseous bridge between the elbow and wrist to facilitate hand function. Surgical formation of the one-bone forearm via a radioulnar synostosis that was first described by Hey-Groves in 1921 [16] is an extreme solution to chronic forearm instability to be used in exceptional circumstances when no other reconstructive options are possible. The largest series to date is that of Peterson et al. [17] who reported in 1995 on the outcome of a heterogeneous group of 19 patients who underwent the procedure post-trauma, after tumour resection or to treat congenital deformity. The complication rate was high especially in the trauma group with an overall non-union rate of 32 %. In 1998, Chen et al. [18] reported their experience of seven patients requiring revision of the un-united one-bone forearm. They advocated abundant bone grafting and plating for rigid fixation in revision cases.

In 1992, Trousdale et al. [19] demonstrated in his review of 20 cases that only 25 % of patients with longitudinal radioulnar dissociation were diagnosed at presentation, and of those with a delayed diagnosis and subsequent treatment, only 20 % had positive outcomes. Although the optimal management of chronic interosseous membrane insufficiency is still evolving, what is certain is that prompt diagnosis optimizes outcome. Careful clinical and radiological examination of the wrist in all significant elbow injuries is strongly advocated to avoid a missed diagnosis. If interosseous membrane lesions are suspected, sonography or magnetic resonance imaging (MRI) should be performed preoperatively. Furthermore, longitudinal stability should be verified intraoperatively—e.g. using the radius pull test—to exclude falsely negative MRI or sonography reports.

It is also important to keep in mind the possibility of an occult Essex-Lopresti lesion even in simple radial head fractures before performing any potentially destabilizing procedure to the forearm such as radial head resection or distal ulnar resection in the case of a Suave-Kapandji procedure. Creating a longitudinal defect in either or both forearm bones, in the presence of an incompetent interosseous, membrane will inevitably exacerbate instability. Furthermore, this case report underlines the important role of the radial head in stabilizing the elbow and forearm not only in the acute but also in the chronic situation. Replacement of the radial head after secondary radial head resection could have prevented this devastating course.
